# Study on Microstructure and Properties of WC Particle-Reinforced FeCoCrNi-Matrix High Entropy Alloy Composites

**DOI:** 10.3390/ma16237380

**Published:** 2023-11-27

**Authors:** Chenglin Zhang, Xian Luo, Liufang Ma, Le Hou, Bin Huang, Rui Hu

**Affiliations:** State Key Laboratory of Solidification Processing, Northwestern Polytechnical University, Xi’an 710072, China; zhangcl0731@mail.nwpu.edu.cn (C.Z.); mlf@mail.nwpu.edu.cn (L.M.); huangbin@nwpu.edu.cn (B.H.); rhu@nwpu.edu.cn (R.H.)

**Keywords:** FeCoCrNi, WC particle, metal-matrix composite, mechanical properties, friction performance

## Abstract

In recent years, high entropy alloy (HEA) matrix composites have undergone rapid development. In this work, the effects of different WC contents (10 wt.%, 20 wt.%, and 30 wt.%) on the microstructure, mechanical properties, and wear resistance of FeCoCrNi HEA matrix composites prepared by spark plasma sintering (SPS) were studied. The results show that the WC–HEA composites are mainly composed of an FCC matrix phase (Ni, Fe) and carbide phases (Cr_7_C_3_, Co_3_W_3_C, WC, etc.). The hardness of the 30 WC–HEA composites was the highest at 459.2 HV, which is 71.2% higher than the 268.3 HV of the pure matrix material. Similarly, the compressive yield strength of the 30 WC–HEA composite was the largest, reaching 1315.1 MPa, which is 112.1% higher than that of the pure matrix material. However, the compression deformation rate of the 30 WC–HEA composite significantly decreased to 16.6%. Under the same dry friction conditions, the addition of an appropriate amount of WC particles can reduce the friction coefficient of the HEA matrix. The wear volume of the composites decreased rapidly with the increase of WC content. The wear volume of 30 WC–HEA was the lowest, only 3.17% of that of the pure matrix material.

## 1. Introduction

In 2004, Yeh and coauthors [1] proposed a theoretical design basis for a multi-principal alloy and named it high entropy alloys (HEA). HEA was defined as containing at least five main elements. In recent years, many researchers have defined quaternary equimolar alloys and medium entropy alloys as high entropy alloys. Therefore, the definition of HEA is not particularly strict [2,3]. As a new type of multi-principal element alloy, HEA have four typical effects, including high entropy effect, lattice distortion effect, slow diffusion effect, and cocktail effect [4]. Among them, high hybrid entropy can make the alloy tend to form a stable single phase solid solution [5]. At present, the most studied alloys in the field of HEA are FCC structured alloys such as Fe, Co, Cr, Ni, and Al. These elements tend to form a simple solid solution structure during the alloying process [6,7], such as FCC or BCC which is attributed to their distinctive mechanical and physical properties [8,9].

With the help of the four effects of HEAs, HEAs can obtain unique microstructure and excellent properties, such as good wear resistance, excellent thermal stability, extremely high ductility, excellent fatigue resistance, and fracture resistance [10,11,12,13,14,15,16,17,18]. The typical high entropy alloy FeCoCrNi has been widely used in many fields, such as hydrogen storage materials, soft magnetic materials, and structural materials due to its excellent tensile properties and low temperature fracture toughness [19,20,21,22,23].

With the further exploration of materials, HEA matrix composites have become a new research direction. HEA matrix composites can combine the excellent plastic deformation ability of the matrix and the high strength and high hardness of the reinforcing phase, which is expected to be widely used in structural parts, wear-resistant materials, and other fields [22,24]. Rogal [25,26] successfully prepared CoCrFeMnNi-5 wt.% Al_2_O_3_ and CoCrFeMnNi-5 wt.% SiC composites by mechanical alloying and hot isostatic pressing. Compared with the high entropy alloy matrix, although some plasticity is sacrificed, the compressive yield strength and hardness of the composites are improved. Yim [27] prepared CoCrFeMnNi-5 wt.% TiC composites by water atomization (WA), mechanical ball milling (MM), and the SPS process. It was found that the compressive yield strength of the composites was improved while the plasticity was almost not damaged. This is because the strain distribution after compression deformation is not limited to the matrix/TiC interface, and the interface does not form stress concentration, so the strength is improved without sacrificing plasticity. Szklarz [28] prepared CoCrFeMnNi-5 wt.% SiC composite by mechanical alloying and hot isostatic pressing. The experimental results showed that SiC nanoparticles not only improve the mechanical properties of the material, but also make it have a certain resistance to chloride corrosion. Wang [29] prepared in situ TiC particle-reinforced FeCoCrNiCu composites by vacuum induction melting. The experimental results showed that the in situ TiC reinforced phase improved the tensile strength of the composite, but the elongation decreased. After adding 10 vol.% TiC reinforced phase, the tensile strength of the composite increased from 560.3 MPa to 705.2 MPa, but the elongation decreased from 53.3% to 23.3%. Li [30] also prepared in situ TiC + SiC particle-reinforced FeCrCoNi composites by vacuum induction melting. The results showed that the FeCrCoNi matrix was composed of an FCC phase, and the in situ particles were uniformly dispersed in the matrix. The addition of the reinforcements increased the ultimate tensile strength of the material from 452 MPa to 783 MPa at room temperature, while the elongation decreased from 70.6% to 28.6%. The improvement of strength can be attributed to the combined effects of thermal mismatch strengthening, grain refinement, and solid solution strengthening [30,31].

At present, the research of HEA matrix composites is still in its infancy. The research on particle-reinforced HEA matrix composites mainly focuses on the in situ formation of TiC particles. There are few studies on HEA matrix composites reinforced by external particles, and the main focus is on the addition of SiC particles. Compared with TiC particles, WC particles have a high hardness and melting point, good wettability with molten metals, and are easier to prepare than TiC. Therefore, it is a reasonable method to use WC particles to strengthen HEA [32]. Zhou [33] prepared WC-AlCrFeCoNi composites by an aerosol method. The results showed that the precipitation of Al during the sintering process makes the HEA matrix change from BCC structure to FCC structure. In addition, WC-AlCrFeCoNi has excellent mechanical properties. The hardness of WC-AlCrFeCoNi with 10% mass fraction was as high as 2160 HV. Khallaf [34] studied the effect of WC addition on the hardness and corrosion behavior of (CrFeCoNi)_1−x_(WC)_x_ (x = 0−20 wt.%) composites. The results showed that the corrosion resistance of the composite was significantly improved with the increase of WC within 20 wt.%, and the hardness at room temperature was also increased from 336.41 HV to 632.48 HV. However, some scholars have proposed that excessive WC would have an opposite effect on wear resistance. Xu and coworkers [35] found, in their study of WC-CrMnFeCoNi coatings, that excessive WC may cause excessive abrasive wear, leading to an increase in the friction coefficient of the coating. In addition, excessive WC causes the aggregation of carbides, accelerating the dissolution and corrosion of the coating.

In a word, although there is currently research on HEA matrix composites, there are relatively few studies on WC-reinforced HEA matrix composites; in particular, the research on wear resistance is quite limited. The reinforcement particles not only have high hardness, but also have excellent anti-wear performance. Therefore, in this work, the FeCoCrNi HEA matrix composites reinforced by WC particles were prepared by SPS technology with excellent mechanical performance [36]. The effects of WC contents of 10 wt.%, 20 wt.%, and 30 wt.% on the microstructure, microhardness, compression performance, and wear resistance of WC–HEA composites were compared and examined.

## 2. Materials and Experiments

### 2.1. Material Preparation

The powder raw materials used in this experiment were mainly Fe, Co, Cr, and Ni metal powders (purity > 99.95%) with particle sizes less than 45 μm and WC particles (purity > 99.9%) with an average particle size of 300 nm. After the FeCoCrNi high entropy alloy powder was prepared according to the raw materials, WC–HEA composite blocks were prepared with different WC contents using FeCoCrNi as the matrix, and FeCoCrNi blocks (HEA) without WC were prepared as a comparison. In the past, the amount of WC particles added to high entropy alloys were usually about 10 wt.% [37]. Through our preliminary experiments, it was found that the brittleness of the sample was too high when the amount of WC added exceeded 30 wt.%. In view of this, in this experiment, the gradient of WC addition was set to 10 wt.%, 20 wt.%, and 30 wt.%, and the prepared composites were denoted as 10 WC–HEA, 20 WC–HEA, and 30 WC–HEA, respectively.

### 2.2. Experimental Process

**Figure 1:**
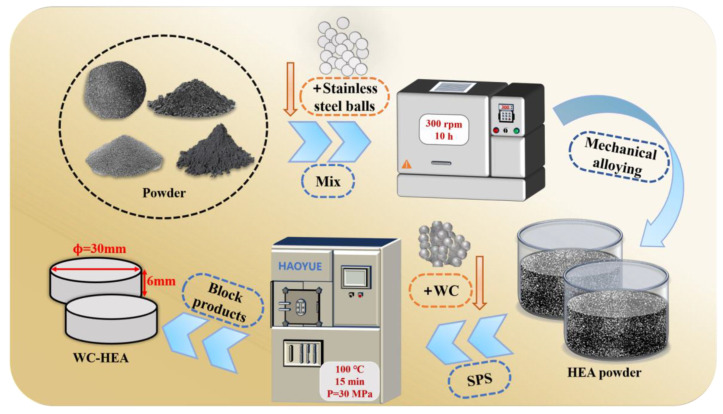
Schematic diagram of the preparation process of the WC–HEA composites.

The preparation process of the composites was: weighing powder → mechanical alloying of matrix powder → high energy ball milling of WC/matrix mixed powder → sintering. The flow chart of the experiment is shown in Figure 1.

Preparation of powders: In order to suppress the reaction between WC powder and pure metal powder during ball milling, WC–HEA composite powder was prepared by a two-step ball milling method. For the HEA matrix, according to the composition ratio of equal atomic ratio, the pure metal powder of Fe, Co, Cr, and Ni was weighed by an electronic balance, and the weighed powder was poured into a vacuum stainless steel tank. According to the ball material ratio of 10:1, an appropriate proportion of stainless steel balls was added. Stainless steel balls and powders only filled half of the vacuum stainless steel tank space to ensure complete mechanical alloying [38]. At the same time, in order to prevent the powder from sticking to the tank, a small amount of anhydrous ethanol was added as a process control agent. HEA powder was prepared by mechanical alloying using a planetary ball mill. The ball mill speed was set at 300 rpm and the time was 10 h. Subsequently, HEA and WC powders were weighed according to the amount of WC added. The second step of ball milling was high energy mixed ball milling, with a ball milling time of 10 h. The addition amount of the HEA and WC powders in the tank only accounted for 50% of the container, thus achieving uniform mixing [38]. Before the start of ball milling, the ball mill tank was placed in a glove box filled with high purity argon as a protective atmosphere. After the ball milling was completed, the powder was poured into a 200 mesh sieve for screening powder, so that the powder size was kept as small and uniform as possible. Powder loading, extraction, and screening were all carried out in the glove box.

Preparation of composite blocks (sintering): The powder was loaded into a high-strength graphite mold in the glove box for sintering. In order to facilitate demolding and prevent a reaction between the powder and the graphite mold, graphite paper was used to separate the contact surface between the mold and the powder. After the powder was loaded, a powder tablet press was used to pre-press the powder, and then sintered and densified using SPS technology. The SPS sintering parameters were 1000 °C/15 min/30 MPa. After the SPS was completed, the pressure was relieved, and the sample was cooled to room temperature in the furnace. The size of the prepared composite sample was approximately Φ 30 × 6 mm.

### 2.3. Microstructural Characterization

A DISCOVER A25 X-ray diffractometer (XRD, Bruker, Billerica, MA, USA) was used to perform phase analysis on the powder samples and sintered blocks after ball milling, and the phases were calibrated based on the position and strength of the diffraction peaks. A Nova^TM^ Nano 450 field emission scanning electron microscope (SEM, FEI, Hillsborough, OR, USA) was used to observe the surface morphology of the original powder, sintered block, and the samples after friction and wear. At the same time, the chemical composition and element distribution of the sample were determined by EDS surface scanning. The phase structure and precipitated phases of the samples were analyzed and characterized by a Talos F200X transmission electron microscope (TEM, Thermo Fisher, Waltham, MA, USA). The surfaces of the samples after the friction and wear experiment were analyzed by an NPFLEX three-dimensional optical profiler (Bruker) to determine the wear volume, depth and width of the samples, so as to quantitatively compare the wear resistance of the samples.

### 2.4. Properties Tests

According to the theoretical density calculation Formulas (1) and (2), the theoretical densities of the HEA matrix and WC–HEA composites were obtained [39]. Then, the true densities of SPS sintered samples after grinding and drying were measured according to the Archimedean principle. The ratio of the true density to theoretical density is the densification of the sample.

(1) $$\rho_{\left(HEAs\right)}=\frac{\sum_{i=1}^{n}C_{i}A_{i}}{\sum_{i=1}^{n}C_{i}A_{i}/\rho_{i}}$$

(2) $$\rho_{\left(WC-HEAs\right)}=\frac{1}{W_{HEAs}/\rho_{HEAs}+W_{WC}/\rho_{WC}}$$

In the formulas, $C_{i}$ is the molar fraction of the *i*-th element, $A_{i}$ is the molar mass of the *i*-th element, $\rho_{i}$ is the density of the *i*-th element (g/cm^3^), and W is the mass fraction.

**Figure 2:**
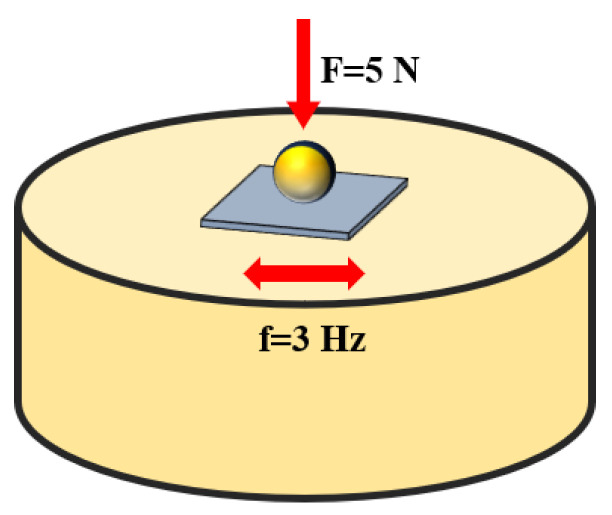
Schematic diagram of the friction and wear test.

In addition, the micro-hardness of the HEA matrix and sintered blocks was measured using the DIIV-1000Z digital micro-Vickers hardness tester (Shangcai Tester Machine, Shanghai, China). A room temperature compression test was conducted on the sample using the Instron-1195 universal testing machine, and the compressive stress–strain curves of the matrix material and sintered blocks were obtained. What is more, before using a three-dimensional optical profilometer, the reciprocating friction and wear tests were conducted on HEA matrix material and sintered blocks using the LMT-100 liquid metal friction and wear testing machine at room temperature (Rtec, San Jose, CA, USA). The contact method for the test was a ball-disc type, using a GCr15 steel ball as the friction pair. The sample was fixed on the load block, as shown in Figure 2. The test load was set to 5 N, the frequency of the load block was 3 Hz, and the wear time was 20 min.

## 3. Results and Discussion

### 3.1. Microstructure of the Matrix Powder

**Figure 3:**
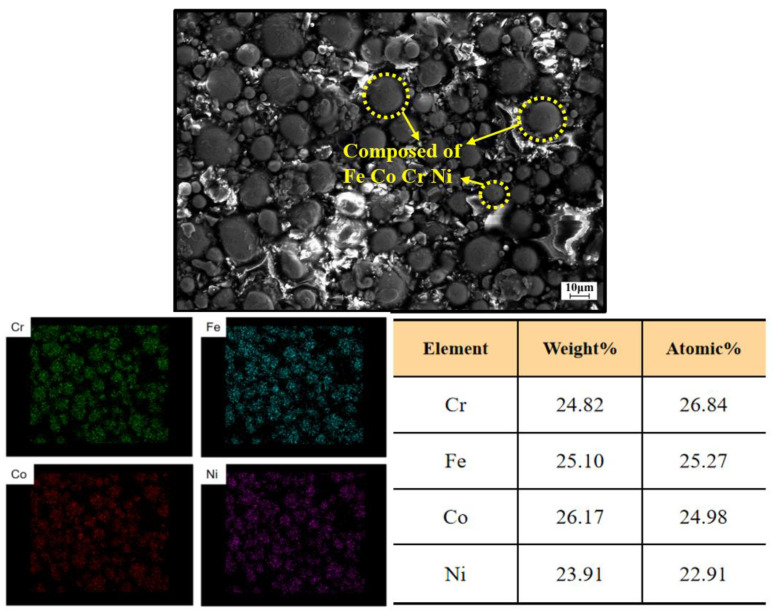
Microstructure and corresponding EDS mapping analysis results of the FeCoCrNi matrix powder.

**Figure 4:**
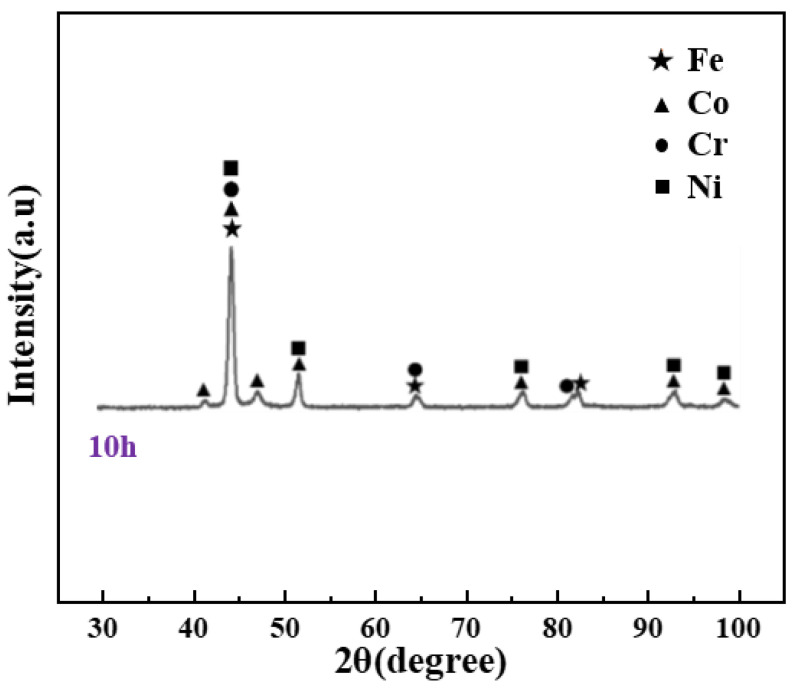
XRD pattern of the FeCoCrNi matrix powder after 10 h of high energy ball milling.

Figure 3 shows the microstructure and corresponding EDS mapping analysis of the FeCoCrNi matrix powder after 10 h of mechanical alloying. It can be seen that the four elements Fe, Co, Cr, and Ni were uniformly mixed, with little difference in mass fraction and atomic number. Figure 4 is the XRD pattern of the matrix powder after 10 h of mechanical alloying. Except for the clear diffraction peaks of the single principal element Co, all other elements showed obvious solid solution alloying phenomenon.

### 3.2. The Effect of WC Content on the Microstructure of Composites

(1) Phase composition analysis

**Figure 5:**
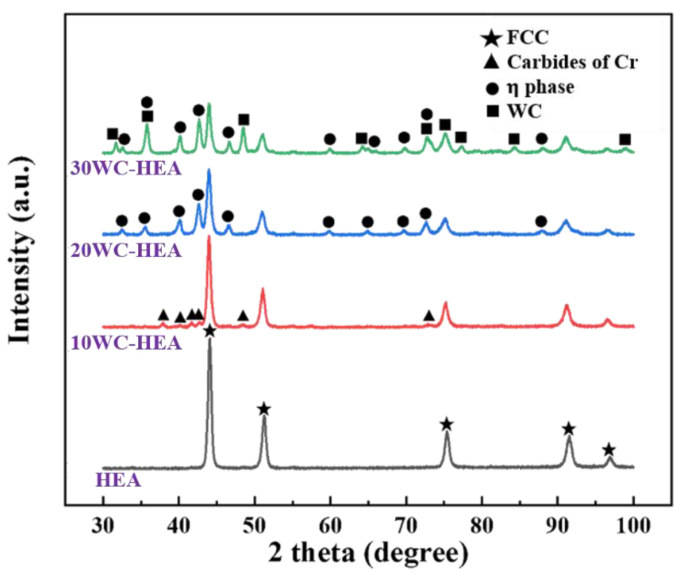
XRD patterns of composites with different WC contents after sintering.

Figure 5 shows XRD patterns of the HEA matrix composite blocks with different WC contents after sintering. The pure HEA matrix powder without WC did not form a single-phase FCC structure before sintering, but a single FCC solid solution structure was formed after SPS, indicating that SPS helped to complete alloying of HEA. The XRD patterns of the HEA matrix composites with different WC contents show that when 10% WC is added, a small number of diffraction peaks of Cr-rich carbides (Cr_23_C_6_, Cr_7_C_3_) appear. When the WC content increases to 20%, Co_3_W_3_C (M_6_C-type η phase) appears. The diffraction peaks of Co_3_W_3_C and Cr_7_C_3_ partially overlap, and the intensities of the diffraction peaks increase with the increase of WC content, indicating an increase in the content of Co_3_W_3_C and Cr_7_C_3_. When the content of WC is 30%, there is an obvious diffraction peak of WC.

The properties of carbide phases in HEA matrix composites are mainly determined by the chemical composition, solidification mode, and external conditions (temperature, time, stress, etc.). For the WC–HEA composites studied, M_23_C_6_, M_7_C_3_, M_6_C, and MC are the four most common types of carbides [40,41,42]. Their amount, size, distribution, and morphology in the matrix will have a significant impact on the properties of the composites.

For M_23_C_6_-type carbides, M in M_23_C_6_ is mainly a Cr element, which is because the diffusion of the C element requires time and distance. Compared with other types of Cr-rich carbides, the C content required to form M_23_C_6_-type carbides is the lowest, so the Cr-rich carbides in the alloy are mainly composed of M_23_C_6_. M_23_C_6_ is an FCC lattice structure, a = 1.066 nm. It can also be found from the XRD patterns that the diffraction peaks of Cr-rich carbides correspond well to M_23_C_6_; M_7_C_3_ carbides. In these kinds of carbides, M is also mainly Cr, and Cr_7_C_3_ carbides are orthorhombic. M_23_C_6_ in the region with high carbon content in the alloy will convert to Cr_7_C_3_; For M_6_C-type carbides (also known as η Phase), when WC is used as reinforcement particles, M_6_C carbides are often formed in the composites. The M_6_C-type carbide is a kind of ternary carbide, namely A_3_B_3_C. The typical type is Co_3_W_3_C with an FCC structure and a = 1.109 nm. M_6_C is more stable than M_23_C_6_ and is one of the important strengthening phases. The results showed that M_23_C_6_-type carbides tend to form in the high Cr region, while M_6_C-type carbides tend to form in the high W region. For an MC-type carbide, in this system, an MC-type carbide mainly refers to the added WC particles. WC is relatively stable and is the most important strengthening phase. The fine, dispersed, and stable WC particles have an obvious strengthening effect on the matrix. In addition, WC also provides carbon atoms for the formation of M_6_C and M_23_C_6_ carbides in the matrix, which is conducive to the formation of other carbides in the system.

(2) Microstructure analysis

**Figure 6:**
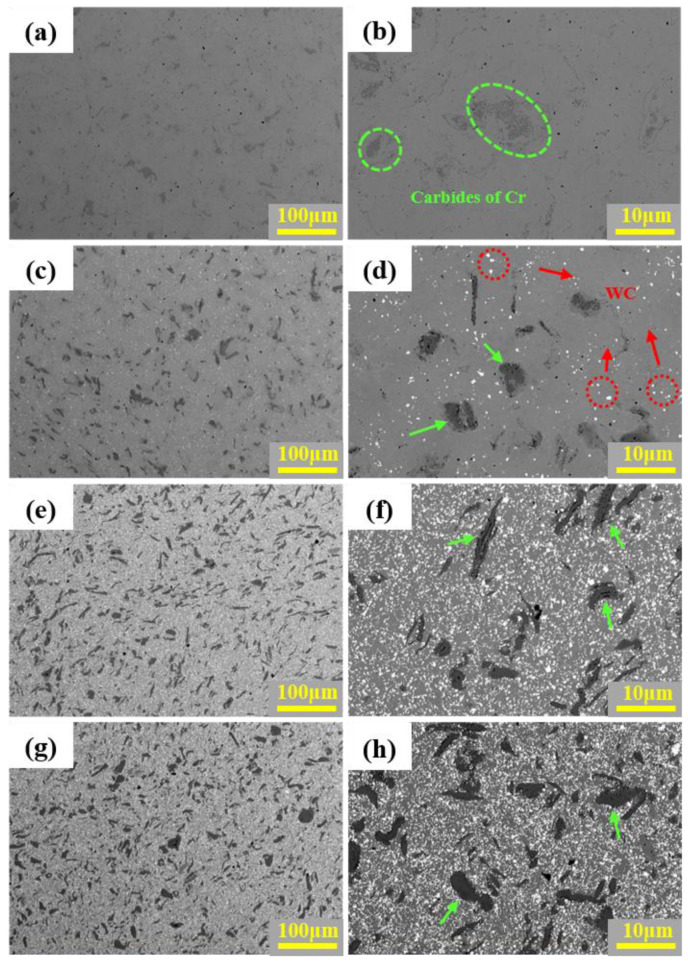
Microstructure of the HEA matrix composites with different WC contents: (**a**) and (**b**) are HEA; (**c**) and (**d**) are 10 WC–HEA; (**e**) and (**f**) are 20 WC–HEA; (**g**) and (**h**) are 30 WC–HEA.

**Figure 7:**
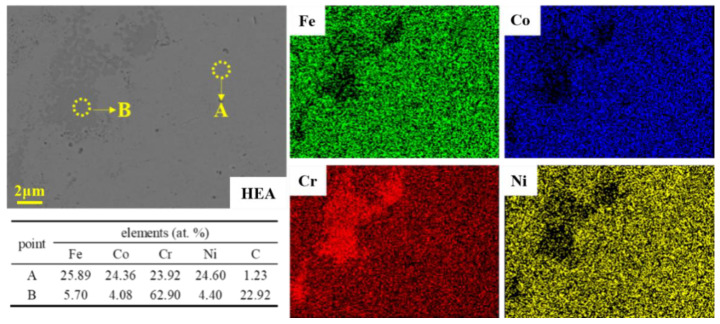
EDS images of the HEA matrix.

Figure 6 shows the microstructures of the HEA matrix composites with different WC contents. It can be seen from Figure 6a,b that, under the backscattered electron mode of SEM, the pure HEA matrix mainly shows a single contrast, with a large number of light gray areas and a small amount of dark gray areas. EDS analysis in Figure 7 shows that the light gray area (region A) is FeCoCrNi HEA with a nearly equal atomic ratio, and the dark gray area (region B) is Cr-rich carbide. Otherwise, 22.92 at. % C is also detected in region B, indicating relatively severe C pollution [43]. This is mainly because the process control agent (C_2_H_6_O) was added in the experiment. In the processes of MA and SPS, the C atoms in C_2_H_6_O were also alloyed and sintered with the metal powders, resulting in a certain degree of C pollution. It can also be found from Figure 6 that when the content of WC is 10%, a small amount of bright white WC particles are dispersed in the HEA matrix (the red arrows), WC particles are agglomerated in a small range, and the dark gray areas rich in Cr increases (the green arrows). This is due to the mutual diffusion of C in WC and Cr in the matrix, resulting in more Cr-rich carbides. As the WC content continues to increase from 20% to 30%, the needle-like Cr-rich region is partially coarsened, the color of the Cr-rich region becomes darker, and the Cr-rich regions become more obvious. At the same time, the white and bright WC particles become finer and more uniform, dispersed in the HEA matrix.

**Figure 8:**
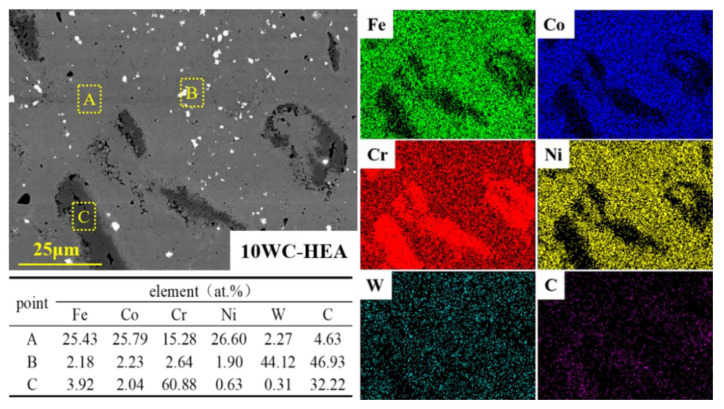
EDS mapping and point analysis results of the 10 WC–HEA composite.

EDS analysis was performed on each region of the 10 WC–HEA sample. In the light gray area (region A) of Figure 8, the atomic ratio of the Fe, Co, and Ni is almost equal (about 25 at.%), and the atomic ratio of Cr (about 15 at.%) is slightly lower than the other three metal elements. The white particles (in region B) in Figure 8 mainly contain W and C elements, which can be determined to be the added WC particles. In the dark gray area (region C) and black dot area, the Cr element has serious segregation, and the atomic ratio of Cr:C at region C is close to 2:1. The combination of EDS composition analysis and XRD detection results indicate that it is Cr_23_C_6_ and Cr_7_C_3_. 

**Figure 9:**
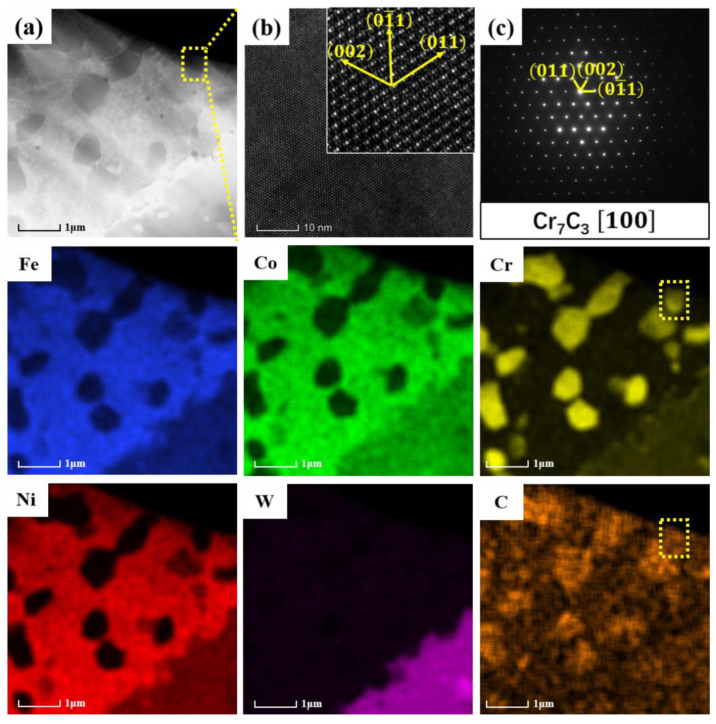
TEM and EDS analysis of the 10 WC–HEA: (**a**) HAADF; (**b**) high-resolution image of the yellow region in (**a**); (**c**) SAED of the yellow region in (**a**).

In order to further verify the crystal structure of the black phase, EDS composition analysis and phase calibration were conducted by TEM at high magnification, as shown in Figure 9. A black irregular polygon particle with a diameter of about 500 nm was selected in the image of Figure 9a. EDS analysis showed that the black-gray particle was composed of Cr and C. In order to determine its crystal structure, high-resolution images and selected-area electron diffraction (SAED) pattern were taken (as shown in Figure 9b,c). Through calibration, it can be seen that the interplanar spacing and interplanar angle of the black particles match well with Cr_7_C_3_, further verifying that the black particles are Cr_7_C_3_.

**Figure 10:**
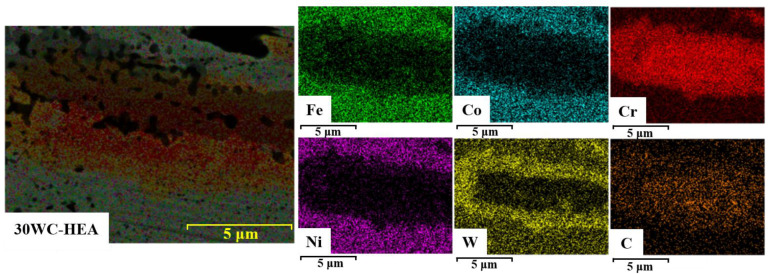
EDS mapping results of the 30 WC–HEA composite.

The EDS mapping results of the dark gray areas in 30 WC–HEA composite are shown in Figure 10. It can be seen that a circle of W rich region is formed near the Cr-rich carbides, which may be related to the mutual transformation between carbides.

**Figure 11:**
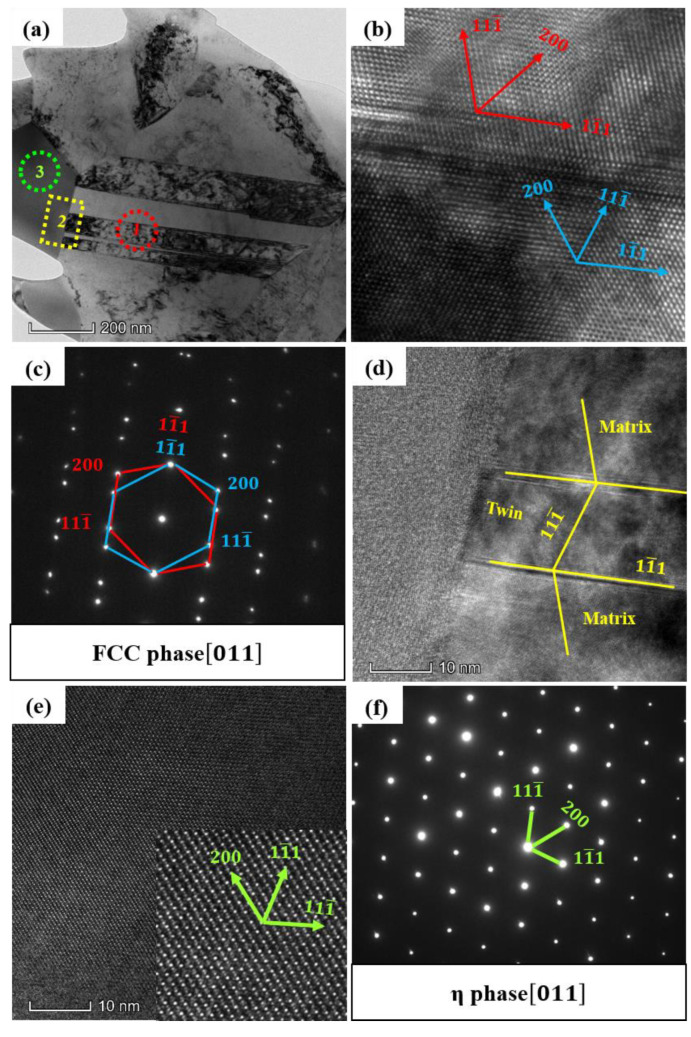
Morphology and structure of the 30 WC–HEA under TEM: (**a**) BF; (**b**,**c**) are high-resolution image and SAED of region 1, respectively; (**d**) high-resolution image of region 2; (**e**,**f**) are high-resolution image and SAED of region 3, respectively.

The microstructure of the 30 WC–HEA composite was further analyzed by TEM, and the results are shown in Figure 11. Figure 11a shows the TEM bright-field images of twin phase (region 1) and precipitated phase (region 3). Figure 11b,c show the high-resolution image and electron diffraction pattern of the twin phase, respectively, and the results show that the interplanar spacing of the twin phase ($1\bar{1}1$) is 2.07 Å, the interplanar spacing of (200) is 1.75 Å, and the interplanar spacing of ($11\bar{1}$) is 2.03 Å. In the standard PDF card, the {111} interplanar spacing of the (Ni, Fe) matrix is 2.08 Å, and the {200} interplanar spacing is 1.80 Å, indicating that the twin phase is basically the same as the matrix atomic arrangement, which is the (Ni, Fe) phase. Figure 11e,f show the high-resolution image and electron diffraction pattern of the precipitated phase, respectively. After calibration, the precipitated phase was identified as Co_3_W_3_C. Figure 11d is a high-resolution image of the interface between the matrix twin and the η phase. The twin phase and the matrix are actually in a twin relationship on the ($1\bar{1}1$) plane, with a 34.55° shift on the ($11\bar{1}$) plane. There is obvious lattice distortion at the twin interface, which affects the atomic arrangement of several atomic planes at the interface and leads to the loss of some atomic planes. The η phase is relatively vague, while the matrix is relatively clear, indicating that there is no obvious orientation relationship between the two. The HEA matrix and Co_3_W_3_C carbides have an FCC structure, which corresponds to the XRD analysis results. 

Combined with Figure 5 and Figure 11, it can be seen that the η phase appears when the WC content is 20%, and continues to exist when the WC content is 30%. This is due to the increased solubility of WC in Co after high temperature sintering, accompanied by the diffusion of W and C atoms in FCC solid solution; the diffusion rate of C is faster than that of W. After cooling, C was partially missing, resulting in an uneven distribution of W and C in the solid solution, resulting in Co_3_W_3_C (η) [44,45,46]. After the formation of the η phase, with the increase of WC content, the η phase also gradually increased. When the WC content was less than 20%, on the one hand, it is because the WC content is not sufficient to support the formation of the η phase and, on the other hand, it is because the formation of carbides such as Cr_7_C_3_ consume some C atoms, so when the WC content is less than 20%, no obvious η phase is found. In addition, the appearance of annealing twins in this series of HEA also proves that they have a lower stacking fault energy [47].

### 3.3. The Effect of WC Content on Properties of Composites

(1) Densification

**Figure 12:**
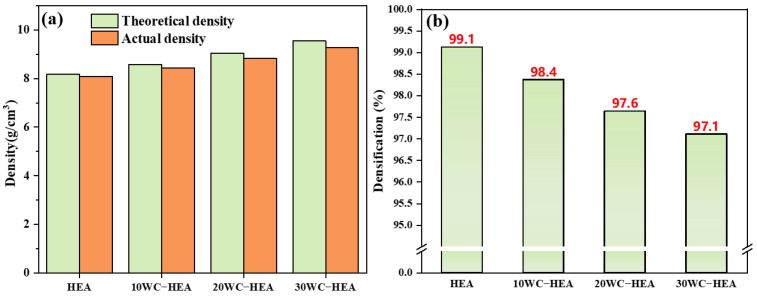
The density (**a**) and densification (**b**) of the HEA matrix composites with different WC contents.

Figure 12 shows the density and densification of the HEA composites with different WC contents calculated by the mixing law Formulas (1) and (2). It can be seen that, with the increase of WC content, the theoretical density and actual density are improved, whereas the actual density is lower than the corresponding theoretical density [31,39]. After SPS at 1000 °C, the densifications of HEA and WC–HEA were 99.1%, 98.4%, 97.6%, and 97.1%, respectively. The densification of the samples gradually decreased with the increase of WC content, which is similar to the densification change trend measured by Zhao [31] on the addition of Al_2_O_3_ particles in CrCoNi matrix. This result may be because the increase of WC leads to the change of carbide and the increase of interface between different phases, which leads to the decrease of material densification. However, in general, the densifications of the HEA matrix composites were above 97%.

(2) Microhardness

**Figure 13:**
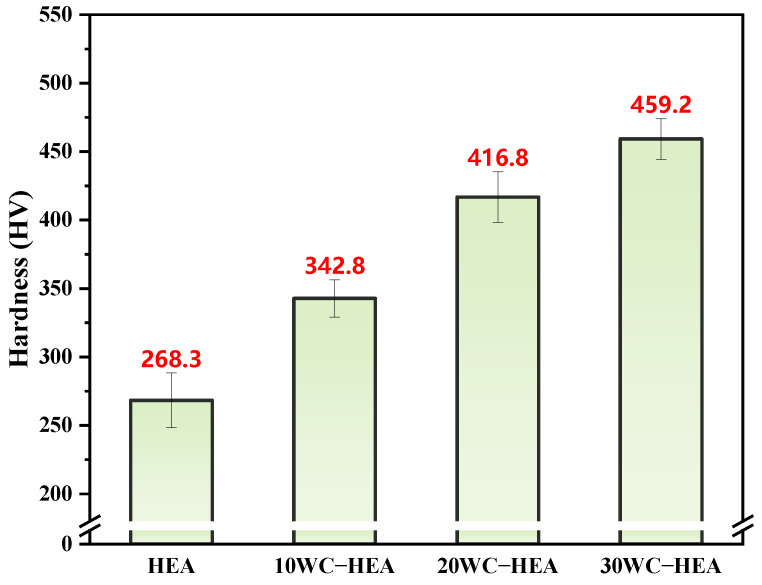
Hardness of HEA matrix composites with different WC contents.

The hardness of the WC–HEA composites with FeCoCrNi as a matrix was tested by a microhardness tester. The test results are shown in Figure 13. It can be seen that the higher the content of WC reinforced phase, the higher the hardness of the composite. For FeCoCrNi matrix HEA composites, the hardness of the pure matrix was only 268.3 HV; when the WC content was 10%, the hardness of 10 WC–HEA composite increased rapidly to 342.8 HV. As the WC content continues to increase, the increasing trend of hardness gradually slows down. When the addition amount of WC was 30%, the hardness reached 459.2 HV.

The strengthening mechanisms of WC particle-reinforced HEA matrix composites mainly include the second phase strengthening of the hard phase itself, the dislocation strengthening introduced by the difference in thermal expansion coefficients between the hard phase and the matrix, the fine grain strengthening of the matrix grains by the hard phase during the ball milling process, and solid solution strengthening caused by diffusion of W and C atoms [48,49].

The hardness of the composites increases with the increase of WC content mainly for two reasons: on the one hand, WC is a common high hardness strengthening phase, with a hardness comparable to that of diamond. Therefore, according to the law of mixtures, the higher the WC content, the higher the hardness of the composites. On the other hand, with the increase of WC content, the concentrations of free W and C dissolved and diffused by WC particles in the HEA matrix also increases, resulting in an increase in precipitated carbide phases, which are generally hard and brittle, thus improving the hardness of the composites. Specifically, the improvement of the hardness of the composites by dissolution of WC particles is the result of a variety of strengthening mechanisms. Firstly, the dissolution of WC particles makes the alloying elements W and C dissolve in the matrix, which play a role in solid solution strengthening. Secondly, Cr_7_C_3_, Co_3_W_3_C and other carbides will produce second phase strengthening. Furthermore, the precipitated carbides will have a fine grain strengthening effect on the HEA matrix, which can also improve the hardness of the composites. 

It is worth noting that, with the increase of WC content, the hardness of HEA composites increases by 27.8%, 21.6%, and 10.2%, respectively, and the growth trend of hardness gradually slows down. The η phase is very important in WC-Co systems and cemented carbide fields [41,50]. In this experiment, the η phase was found when the WC content was added to 20%. When the WC content increased from 20% to 30%, the hardness increment of the HEA was relatively small and did not increase linearly. With the increase of WC content, the η phase increased. This shows that compared with the WC phase, the η had a lower effect on improving the hardness of HEA matrix composites, resulting in the weakening of the strengthening effect of WC particles with the increase of WC content.

(3) Room temperature compressive properties

**Figure 14:**
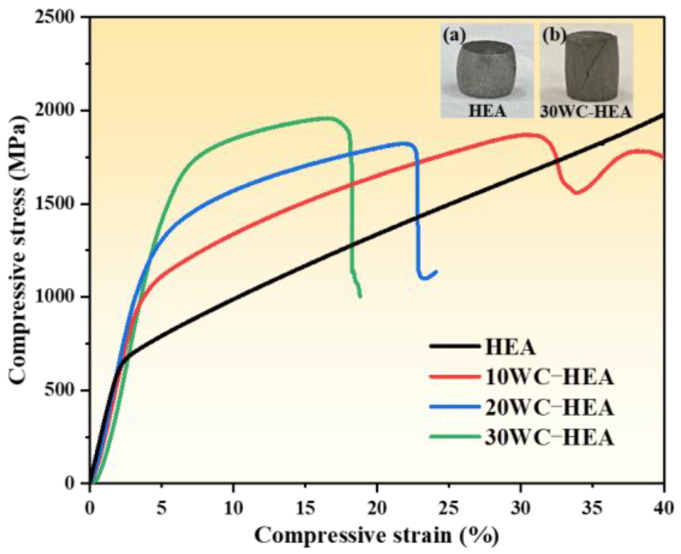
Compressive stress–strain curves of HEA matrix composites with different WC contents.

Figure 14 shows room temperature compressive stress–strain curves of the HEA matrix composites with different WC contents. The compressive yield strength (σ_ys_), ultimate compressive strength (σ_p_), and compression ratio (δ_p_) of the sintered blocks are shown in Table 1. From Figure 14 and Table 1, it can be seen that the compressive yield strength of the sintered sample increases with the increase of WC content, while the compression rate decreases with the increase of WC content. The compressive properties of the sintered blocks are directly related to the phase composition and microstructure of the samples. The fine and dispersed hard phases can hinder the dislocation movement and improve the strength, but the agglomerated Cr-rich regions will cause local stress concentration, resulting in a sharp decline in plasticity.

Combined with the hardness variation pattern of the samples, it can be seen that the compressive yield strength and hardness of the composite exhibit a consistent trend with the WC content. The main factors affecting the hardness are the relative content of WC, Cr-rich phase, η phase, and the grain size of the matrix, and the compressive performance is also closely related to these factors. For FeCoCrNi pure matrix, the plasticity of the sample was very good, and the compression ratio reached over 40%. The upper right corner (a) of Figure 14 shows the macroscopic morphology of the HEA matrix after compression. The matrix exhibits a typical waist drum shape after the compression. The material does not break due to its excellent plasticity. The compressive yield strength of the HEA matrix prepared by SPS was 619.9 MPa, while that of the HEA prepared by most of the arc melting methods was just 500 MPa and below [51,52], indicating that short-time ball milling can also obtain HEAs with excellent mechanical performance. With the increase of WC content, the yield strength gradually increased to over 1000 MPa. When the WC content increase to 30%, the yield strength of the 30 WC–HEA composite reached 1315.1 MPa, but the compression rate rapidly decreases to 16.6%. The upper right corner (b) of Figure 14 shows that the 30 W-HEA is fractured along the inclined plane with an angle of about 45° to the axis after compression, indicating that failed by shear stress. In addition, the 30 WC–HEA sample also broke into some powders after compression, which is the result of the sharp decrease in the plasticity of HEA caused by the excessive addition of WC particles. The macroscopic morphology of the 10 WC–HEA after compression is similar to that of the HEA matrix, which is drum-shaped. The macroscopic morphology of the 20 WC–HEA after compression was similar to that of the 30 WC–HEA, which was inclined and fractured along the axis after compression. Overall, with the increase of WC content, WC, Cr-rich phase, η phase, and other strengthening phases gradually reduced the plasticity of the HEA, and the material gradually changed from unbroken to brittle fracture after compression.

Based on the hardness and compression performance, it can be seen that the 20 WC–HEA composite reinforced with 20 wt.% WC particles has good comprehensive mechanical properties.

(4) Friction and wear properties

In the research on the wear properties of HEA, most of them increase the hardness and wear resistance of the alloys by adding metal elements for solid solution strengthening and precipitation strengthening, while there are few studies that improve their wear resistance by adding hard particles such as WC. According to the above results, adding WC particles to HEA can achieve higher strength and hardness. Therefore, on this basis, this paper further studied the influence of WC particles on the wear resistance of the composites.

**Figure 15:**
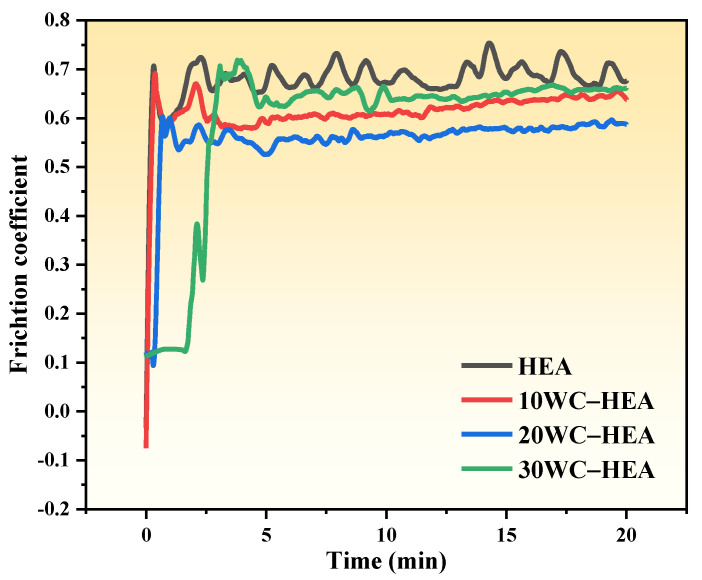
Friction coefficient curves of HEA matrix composites with different WC contents.

The friction coefficient curves of the WC–HEA composites are shown in Figure 15. For the FeCoCrNi pure matrix, its friction coefficient was unstable and fluctuated greatly during the friction process. As the friction process progressed, the final calculated average friction coefficient was 0.68. The friction coefficient of the composites with WC particles added was relatively stable during the wear process, without significant fluctuations. With the increase of WC content, the average friction coefficient of the composite decreased from 0.68 of the pure HEA matrix to 0.61 of the 10 WC–HEA composite, then continued to decrease to 0.55 of the 20 WC–HEA composite, and then increased to 0.60 of the 30 WC–HEA composite. The size of the friction coefficient is mainly related to the surface roughness of the sample. From the results shown in Figure 15, WC helps to reduce the surface roughness and friction coefficient of HEA.

**Figure 16:**
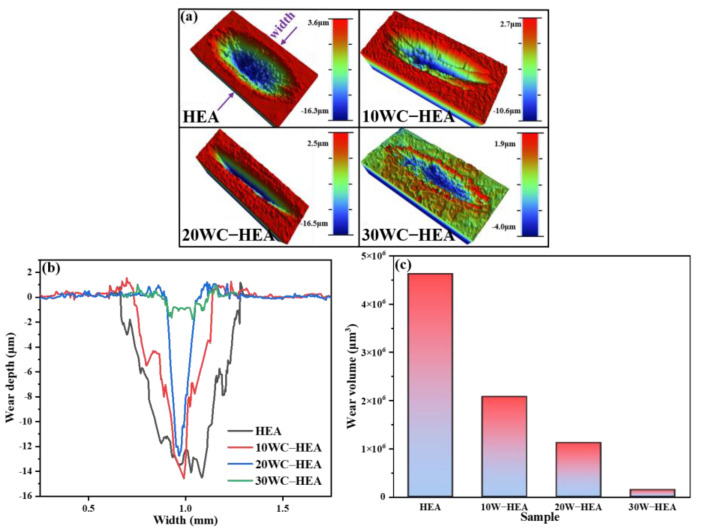
Wear properties of HEA matrix composites with different WC contents: (**a**) 3D topography; (**b**) wear width and depth; (**c**) wear volume.

The surface of HEA matrix composites with different WC contents after wear were tested and analyzed by three-dimensional profilometer. The results are shown in Figure 16. It can be seen from Figure 16a that the surface of the FeCoCrNi matrix has deep and wide grinding cracks. With the addition of WC, the surface of the matrix appears furrows, and the furrows on the 10 WC–HEA sample are the most obvious, indicating that abrasive wear occurs. Under the action of load, the surface material of the sample is worn into abrasive particles. Figure 16b is the comparison of the width and depth of the wear scar. From Figure 16b, it can be seen that from the width of the wear scar, HEA > 10 WC–HEA > 20 WC–HEA > 30 WC–HEA, that is, the depth and width of the grinding pit decrease with the increase of WC content. From the perspective of wear scar depth, with the increase of WC content, the wear scar depth is inversely proportional to the WC content. Figure 16c is the wear volume of samples with different WC contents under the same conditions. In Figure 16c, with the increase of WC content, the corresponding wear volume decreases gradually. It shows that the increase of WC improves the wear resistance of FeCoCrNi matrix to a certain extent. Wear loss is an important index to evaluate the wear resistance of materials. The smaller the wear loss, the better the wear resistance of materials. Compared with the wear volume of pure FeCoCrNi matrix (4.63 × 10^6^ μm^3^), the wear volume of 30 WC–HEA composite is reduced to 1.47 × 10^5^ μm^3^, indicating that the 30 WC–HEA composite has better wear resistance.

**Figure 17:**
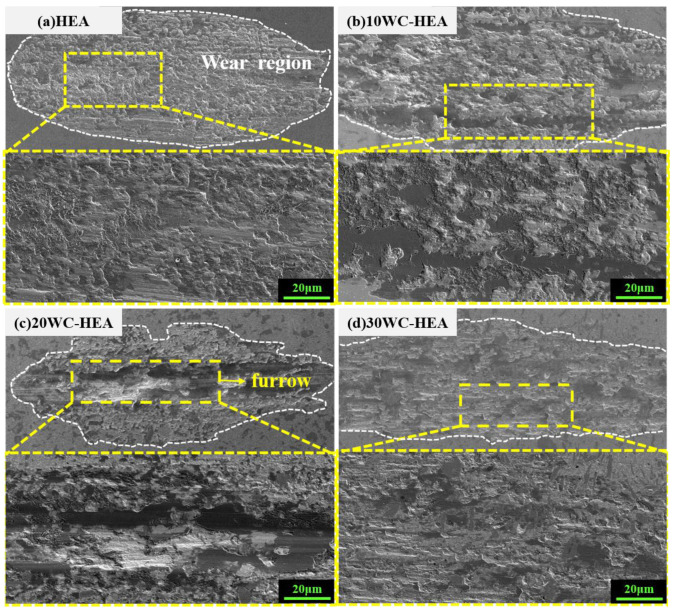
Surface SEM morphology of HEA matrix composites with different WC contents: (**a**) HEA; (**b**) 10 WC–HEA; (**c**) 20 WC–HEA; (**d**) 30 WC–HEA.

Figure 17 shows the surface SEM morphology of the HEA matrix composites with different WC contents after wear. Many adhesive wear areas and delaminations can be observed in the samples. The surface of the FeCoCrNi pure matrix after wear is a regular elliptical shape, and its wear form is mainly adhesive wear that often occurs in plastic materials. The surface of the 20 WC–HEA composite shows obvious furrows after wear, which is due to the generation of a certain amount of hard particles such as WC and Cr_7_C_3_ during the grinding process. The abrasive grains accumulate on the wear surface along the wear trajectory, causing different degrees of damage to the material surface and forming furrows. It can be seen from Figure 17a–c that the wear mechanism of the HEA gradually changed from adhesive wear to abrasive wear with the increase of WC content from 0 to 20%. This is consistent with Xu’s results [35]. Abrasive wear easily accelerates the wear rate in local areas. For 30 WC–HEA composites, compared with Figure 17a–c, the plastic deformation area generated by the worn surface is smaller, without obvious protrusions or grooves, which reflects that the better wear resistance of the composite with 30% WC. In addition, Figure 17 shows that, with the increase of WC content, adhesive delamination (the dark gray flake area in which Figure 17) appears on the sample surface, which may be due to the local temperature increasing during the friction process. A layer of oxide film forms on the metal surface, and then the oxide film becomes soft or peels off, which plays a lubricating role to a certain extent, reducing the friction coefficient of the sample and resulting in delamination.

Combined with Figure 16 and Figure 17, the smaller wear volume and relatively smooth friction surface morphology of WC–HEA composites indicate that the HEA with the addition of WC particles have better wear resistance under the same dry friction conditions. Because when WC particles are added, WC exists in the form of a hard phase in the HEA matrix. WC particles have high hardness, good wear resistance, strong resistance to deformation during friction, and are less prone to plastic deformation, resulting in increased friction resistance and stronger wear resistance of the composites. With the increase of WC content, the wear mechanism of HEA matrix composites changes from adhesive wear to abrasive wear, and the formation of second phases such as Cr-rich and Co_3_W_3_C carbides in the composites also reduces the wear volume, which is beneficial to the improvement of wear resistance of HEA matrix. When the WC content increases to 30%, the WC content is too much, resulting in an excessive amount of the Cr-rich phase being generated in the composite, leading to more prominent interface problems, affecting the bonding between the hard phase and the matrix, resulting in a poor overall lubrication effect and increased friction coefficient of the composite. However, overall, compared with the Cr-rich phase, the distribution of WC particles in the 30 WC–HEA sample is more dispersed, and the effect of WC is more prominent. Although the friction coefficient rises slightly, the friction and wear amount of 30 WC–HEA composite is the lowest among the tested samples, indicating that WC plays an important role in improving the wear resistance of the HEA matrix.

## 4. Conclusions

In this work, WC-FeCoCrNi composites were prepared by the SPS process. The effects of WC content on the microstructure, mechanical properties, and wear resistance of WC–HEA composites were studied. The main conclusions are as follows:

(1) A single-phase FCC solid solution structure was formed after SPS sintering of the HEA pure matrix powder, which did not appear before sintering, indicating that SPS is helpful for HEA to complete alloying.
(2) The sintered WC–HEA composite is mainly composed of an FCC matrix phase (Ni, Fe) and carbide phases (Cr_7_C_3_, Co_3_W_3_C, WC, etc.). With the increase of WC content, the distribution of WC is dispersed, and the content of Cr_7_C_3_ and Co_3_W_3_C increases gradually. Some needle-like Cr-rich phases in the FCC matrix coarsen into flakes.
(3) Although the addition of WC reduces the densification of the composite to a certain extent, it significantly improves the hardness and compressive yield strength of the matrix. The microhardness of the 30 WC–HEA composite is the highest, at 459.2 HV, which is 71.2% higher than that of the pure matrix material. The compressive yield strength of the 30 WC–HEA composite is the highest, 112.1% higher than that of the pure matrix. However, the plasticity of the 30 WC–HEA composite deteriorated, and the compression deformation rate seriously decreased to 16.6%.
(4) Under the same dry friction conditions, the addition of an appropriate amount of WC particles can reduce the friction coefficient of the HEA matrix. In addition, the wear volume of the WC–HEA composite decreases rapidly with the increase of WC content. The wear volume of the 30 WC–HEA composite is 1.47 × 10^5^ μm^3^, accounting for only 3.17% of the pure matrix material, indicating that the HEA with WC particles have better wear resistance.

## Figures and Tables

**Table 1:** Compressive properties of HEA matrix composites with different WC contents.

Sample	σ_ys_ (MPa)	σ_p_ (MPa)	δ_p_ (%)
HEA	619.9	1978.0	>40.0
10 WC–HEA	914.0	1870.9	30.4
20 WC–HEA	1012.9	1823.4	21.8
30 WC–HEA	1315.1	1959.5	16.6

## Data Availability

All data are provided in the article.

## References

[1] Yeh J.W., Chen S.K., Lin S.J., Gan J.Y., Chin T.S., Shun T.T., Tsau C.H., Chang S.Y. (2004). Nanostructured High-Entropy Alloys with Multiple Principal Elements: Novel Alloy Design Concepts and Outcomes. Adv. Eng. Mater..

[2] Gao M.C., Yeh J.-W., Liaw P.K., Zhang Y. (2016). High-Entropy Alloys: Fundamentals and Applications.

[3] Miracle D.B., Senkov O.N. (2017). A critical review of high entropy alloys and related concepts. Acta Mater..

[4] Tsai M.-H., Yeh J.-W. (2014). High-Entropy Alloys: A Critical Review. Mater. Res. Lett..

[5] Tong C.-J., Chen Y.-L., Chen S.-K., Yeh J.-W., Shun T.-T., Tsau C.-H., Lin S.-J., Chang S.-Y. (2005). Microstructure Characterization of Al_x_CoCrCuFeNi High-Entropy Alloy System with Multiprincipal Elements. Metall. Mater. Trans. A.

[6] Sathiaraj G.D., Ahmed M.Z., Bhattacharjee P.P. (2016). Microstructure and texture of heavily cold-rolled and annealed fcc equiatomic medium to high entropy alloys. J. Alloys Compd..

[7] Sathiyamoorthi P., Basu J., Kashyap S., Pradeep K.G., Kottada R.S. (2017). Thermal stability and grain boundary strengthening in ultrafine-grained CoCrFeNi high entropy alloy composite. Mater. Des..

[8] Ye Y.F., Wang Q., Lu J., Liu C.T., Yang Y. (2016). High-entropy alloy: Challenges and prospects. Mater. Today.

[9] Senkov O.N., Wilks G.B., Miracle D.B., Chuang C.P., Liaw P.K. (2010). Refractory high-entropy alloys. Intermetallics.

[10] Liu W.H., Yang T., Liu C.T. (2018). Precipitation hardening in CoCrFeNi-based high entropy alloys. Mater. Chem. Phys..

[11] Chuang M.-H., Tsai M.-H., Wang W.-R., Lin S.-J., Yeh J.-W. (2011). Microstructure and wear behavior of Al_x_Co_1.5_CrFeNi_1.5_Ti_y_ high-entropy alloys. Acta Mater..

[12] Zou Y., Ma H., Spolenak R. (2015). Ultrastrong ductile and stable high-entropy alloys at small scales. Nat. Commun..

[13] Wu Y.D., Cai Y.H., Wang T., Si J.J., Zhu J., Wang Y.D., Hui X.D. (2014). A refractory Hf_25_Nb_25_Ti_25_Zr_25_ high-entropy alloy with excellent structural stability and tensile properties. Mater. Lett..

[14] Deng Y., Tasan C.C., Pradeep K.G., Springer H., Kostka A., Raabe D. (2015). Design of a twinning-induced plasticity high entropy alloy. Acta Mater..

[15] Li D., Li C., Feng T., Zhang Y., Sha G., Lewandowski J.J., Liaw P.K., Zhang Y. (2017). High-entropy Al_0.3_CoCrFeNi alloy fibers with high tensile strength and ductility at ambient and cryogenic temperatures. Acta Mater..

[16] Gludovatz B., Hohenwarter A., Catoor D., Chang E.H., George E.P., Ritchie R.O. (2014). A fracture-resistant high-entropy alloy for cryogenic applications. Science.

[17] Tang Z., Yuan T., Tsai C.-W., Yeh J.-W., Lundin C.D., Liaw P.K. (2015). Fatigue behavior of a wrought Al_0.5_CoCrCuFeNi two-phase high-entropy alloy. Acta Mater..

[18] Hemphill M.A., Yuan T., Wang G.Y., Yeh J.W., Tsai C.W., Chuang A., Liaw P.K. (2012). Fatigue behavior of Al_0.5_CoCrCuFeNi high entropy alloys. Acta Mater..

[19] Kunce I., Polanski M., Bystrzycki J. (2014). Microstructure and hydrogen storage properties of a TiZrNbMoV high entropy alloy synthesized using Laser Engineered Net Shaping (LENS). Int. J. Hydrog..

[20] Zhang Y., Zuo T., Cheng Y., Liaw P.K. (2013). High-entropy Alloys with High Saturation Magnetization, Electrical Resistivity and Malleability. Sci. Rep..

[21] Gali A., George E.P. (2013). Tensile properties of high- and medium-entropy alloys. Intermetallics.

[22] Sun X., Zhang H., Lu S., Ding X., Wang Y., Vitos L. (2017). Phase selection rule for Al-doped CrMnFeCoNi high-entropy alloys from first-principles. Acta Mater..

[23] Lucas M.S., Wilks G.B., Mauger L., Muñoz J.A., Senkov O.N., Michel E., Horwath J., Semiatin S.L., Stone M.B., Abernathy D.L. (2012). Absence of long-range chemical ordering in equimolar FeCoCrNi. Appl. Phys. Lett..

[24] Yan Y.-F., Kou S.-Q., Yang H.-Y., Shu S.-L., Shi F.-J., Qiu F., Jiang Q.-C. (2023). Microstructure-based simulation on the mechanical behavior of particle reinforced metal matrix composites with varying particle characteristics. J. Mater. Res. Technol..

[25] Rogal Ł., Kalita D., Litynska-Dobrzynska L. (2017). CoCrFeMnNi high entropy alloy matrix nanocomposite with addition of Al_2_O_3_. Intermetallics.

[26] Rogal Ł., Kalita D., Tarasek A., Bobrowski P., Czerwinski F. (2017). Effect of SiC nano-particles on microstructure and mechanical properties of the CoCrFeMnNi high entropy alloy. J. Alloys Compd..

[27] Yim D., Sathiyamoorthi P., Hong S.-J., Kim H.S. (2019). Fabrication and mechanical properties of TiC reinforced CoCrFeMnNi high-entropy alloy composite by water atomization and spark plasma sintering. J. Alloys Compd..

[28] Szklarz Z., Lekki J., Bobrowski P., Szklarz M.B., Rogal Ł. (2018). The effect of SiC nanoparticles addition on the electrochemical response of mechanically alloyed CoCrFeMnNi high entropy alloy. Mater. Chem. Phys..

[29] Wang H., Wu Z., Wu H., Zhu H., Tang W. (2021). In Situ TiC Particle-Reinforced FeCoCrNiCu High Entropy Alloy Matrix Composites by Induction Smelting. Trans. Indian Inst. Met..

[30] Li Y.-l., Zhao Y., Shen L., Wu H., Zhu H.-G. (2020). Microstructure and mechanical properties of in situ (TiC + SiC)/FeCrCoNi high entropy alloy matrix composites. J. Iron Steel Res. Int..

[31] Zhao A.W., Luo X., Ye Z.L., Guo X., Huang B., Lu W.J., Li P.T., Yang Y.Q. (2021). Microstructure and mechanical properties of novel CrCoNi–Al_2_O_3P_ medium entropy alloy-matrix composites. Intermetallics.

[32] Bartkowski D., Bartkowska A., Jurči P. (2021). Laser cladding process of Fe/WC metal matrix composite coatings on low carbon steel using Yb: YAG disk laser. Opt. Laser Technol..

[33] Zhou P.L., Xiao D.H., Zhou P.F., Yuan T.C. (2018). Microstructure and properties of ultrafine grained AlCrFeCoNi/WC cemented carbides. Ceram. Int..

[34] Khallaf A.H., Bhlol M., Dawood O.M., Ghayad I.M., Elkady O.A. (2022). Effect of tungsten carbide (WC) on electrochemical corrosion behavior, hardness, and microstructure of CrFeCoNi high entropy alloy. J. Eng. Appl. Sci..

[35] Xu B., Zhou Y., Liu Y., Hu S., Zhang G. (2022). Effect of different contents of WC on microstructure and properties of CrMnFeCoNi high-entropy alloy-deposited layers prepared by PTA. J. Mater. Res..

[36] Oketola A., Jamiru T., Adegbola A.T., Ogunbiyi O., Rominiyi A.L., Smith S. (2023). Spark plasma sintering of ceramic-reinforced binary/ternary nickel and titanium metal matrix composites: Mechanical properties, microstructure, and densification—A review. J. Alloys Metall. Syst..

[37] Zhou R., Chen G., Liu B., Wang J., Han L., Liu Y. (2018). Microstructures and wear behaviour of (FeCoCrNi)_1-x_(WC)_x_ high entropy alloy composites. Int. J. Refract. Met. Hard Mater..

[38] Prică C.-V., Marinca T.F., Popa F., Sechel N.A., Isnard O., Chicinaş I. (2016). Synthesis of nanocrystalline Ni_3_Fe powder by mechanical alloying using an extreme friction mode. Adv. Powder Technol..

[39] Sharma A., Oh M.C., Ahn B. (2020). Microstructural evolution and mechanical properties of non-Cantor AlCuSiZnFe lightweight high entropy alloy processed by advanced powder metallurgy. Mater. Sci. Eng. A.

[40] Liu L., Zhang Y., Han J., Wang X., Jiang W., Liu C.-T., Zhang Z., Liaw P.K. (2021). Nanoprecipitate-Strengthened High-Entropy Alloys. Adv. Sci..

[41] Peng Y., Zhang W., Li T., Zhang M., Liu B., Liu Y., Wang L., Hu S. (2020). Effect of WC content on microstructures and mechanical properties of FeCoCrNi high-entropy alloy/WC composite coatings by plasma cladding. Surf. Coat. Technol..

[42] Lin D., Xi X., Li X., Hu J., Xu L., Han Y., Zhang Y., Zhao L. (2022). High-temperature mechanical properties of FeCoCrNi high-entropy alloys fabricated via selective laser melting. Mater. Sci. Eng. A.

[43] Marinca T.F., Chicinaş H.F., Neamţu B.V., Popa F., Chicinaş I. (2017). Reactive spark plasma sintering of mechanically activated α-Fe_2_O_3_/Fe. Ceram. Int..

[44] Ji G.J., Wei M.X., Wang D.F. (2017). Effect of tungsten carbide particle size on the phase of WC-CoCr powder and coating. Therm. Spray. Technol. China.

[45] Ma N., Guo L., Cheng Z., Wu H., Ye F., Zhang K. (2014). Improvement on mechanical properties and wear resistance of HVOF sprayed WC-12Co coatings by optimizing feedstock structure. Appl. Surf. Sci..

[46] Xu M., Liu X., Lu H., Wang H., Zhao Z., Hou C., Han T., Song X. (2023). Increase of specific interfacial coherence in nanocrystalline ceramic-metal composites. Compos. B. Eng..

[47] Li H., Xia S., Zhou B., Chen W., Hu C. (2010). The dependence of carbide morphology on grain boundary character in the highly twinned Alloy 690. J. Nucl. Mater..

[48] Arsenault R.J., Wang L., Feng C.R. (1991). Strengthening of composites due to microstructural changes in the matrix. Acta Mater..

[49] Starink M.J., Wang P., Sinclair I., Gregson P.J. (1999). Microstructure and strengthening of Al–Li–Cu–Mg alloys and MMCs: I. Analysis and Modelling of Microstructural changes. Acta Mater..

[50] Chen C., Huang B., Liu Z., Li Y., Zou D., Liu T., Chang Y., Chen L. (2023). Additive manufacturing of WC-Co cemented carbides: Process, microstructure, and mechanical properties. Addit. Manuf..

[51] Zhang W., Liaw P.K., Zhang Y. (2018). Science and technology in high-entropy alloys. Sci. China Mater..

[52] Salishchev G.A., Tikhonovsky M.A., Shaysultanov D.G., Stepanov N.D., Kuznetsov A.V., Kolodiy I.V., Tortika A.S., Senkov O.N. (2014). Effect of Mn and V on structure and mechanical properties of high-entropy alloys based on CoCrFeNi system. J. Alloys Compd..

